# Reactivation of *Coccidioides immitis* in a Prosthetic Knee after Initiation of Chemotherapy

**DOI:** 10.1155/2021/3964465

**Published:** 2021-12-21

**Authors:** Zachary Ciochetto, Maria Georgen, Adam Hadro, Lauren Jurkowski, Kimberly Ridolfi, Adam Wooldridge, Nathan Gundacker, Javeria Haque

**Affiliations:** ^1^Medical College of Wisconsin Affiliated Hospitals, Milwaukee, WI, USA; ^2^Zablocki VA Medical Center, Milwaukee, WI, USA

## Abstract

*Coccidioides* is an endemic fungus in the Southwestern United States and Central and South America. Coccidioidomycosis primary infections are typically of the lung with an asymptomatic or self-limiting course. Some infections disseminate to other parts of the body and a few can remain latent for many years. Reactivation of latent fungal disease can occur following an insult to the host immune system. Here, we describe a case of a 76-year-old Caucasian male patient who moved from California to Wisconsin with a history of coccidioidomycosis infection of the left knee that reactivated decades later in his prosthetic knee shortly after being initiated on ibrutinib (Imbruvica), a Bruton tyrosine kinase (BTK) inhibitor, for chronic lymphocytic leukemia (CLL)/small lymphocytic lymphoma (SLL). There have been some case reports regarding coccidioidomycosis infections after initiating ibrutinib therapy but none with a 50 year latency period before reactivation. Readers will learn the immunological effects of ibrutinib on the hosts' innate and adaptive immunity and its role in putting the host at risk for invasive fungal infections. We also review the literature and data on treatment regimens and recommendations based on current guidelines.

## 1. Introduction

Coccidioidomycosis, also known as Valley Fever, is an uncommon infection in the upper Midwest as it is endemic to the southwestern United States and Central and South America. Infections are generally acquired from inhalation of arthroconidia, the spores of the dimorphic organism *Coccidioides immitis,* whereas those acquired elsewhere are from *Coccidioides posadasii*. This fungus causes asymptomatic or mild infections in most patients with some developing acute or subacute pulmonary infections 1–3 weeks after inoculation. An estimated 5–10% of patients develop residual pulmonary disease consisting of nodules or cavitary lesions and about 1% develop disseminated disease. Extrapulmonary sites typically involve the skin and skeletal system [1].

Disruption in the innate immunity with reduced activation of neutrophils by cytokines and INF-y, as well as poor T-cell response and function, can lead to disseminated fungal infections [2]. Patients with CLL/SLL develop a secondary immunodeficiency through lymphocyte dysfunction that is further compounded by immunomodulating treatment therapies, such as ibrutinib, a BTK inhibitor. BTK is important in B-cell receptor signaling maturation and activation. It also has been observed in the pathways activating innate and adaptive immunities with key targets being neutrophils, macrophages, and monocytes. Through BTK inhibition, ibrutinib dysregulates downstream T-cell signaling and maturation, thus leading to ineffective Th2 effector cells, predisposing hosts to invasive fungal infections (IFIs) such as invasive aspergillosis, cryptococcus, and Pneumocystis jiroveci pneumonia (PJP) [3], and as in our case, coccidioidomycosis.

Herein, we present a report of a patient with reactivation of *Coccidioides immitis* knee prosthetic joint infection (PJI) three months after initiation of ibrutinib for CLL/SLL after a 50-year latency period. There are only a few case reports [4, 5] regarding reactivation of articular infection; however, none are described with such a prolonged latency period. We further present relevant literature available thus far on the topic and discuss immunologic mechanisms that may be involved in the fungal pathogenesis in such patients.

## 2. Case Presentation

A 75-year-old male with history of CLL/SLL presented to the emergency department (ED) with complaints of left knee pain and swelling for one week. Patient had undergone left total knee arthroplasty (TKA) for osteoarthritis twelve years prior without post-operative complications. Three months prior to presentation, he was started on ibrutinib for his CLL/SLL due to worsening anemia, leukocytosis, and lymphadenopathy. In the ED, his physical exam was notable for a left knee effusion with tenderness and limited range of motion. His effusion was aspirated, and synovial fluid analysis revealed a total WBC count of 6,404 cells/mm^3^ with 53% monocytes, 29% neutrophils, and 19% lymphocytes. On presentation, his WBC was 7.4 × 10^9^/L (normal 4–12 × 10^9^/L), with 57% lymphocytes and 30% neutrophils, with a hemoglobin of 9.6 g/dl (normal male 13.5–17.5 g/dL) and platelet count of 131 × 10^9^/L (normal 150–450 × 10^9^/L). His erythrocyte sedimentation rate (ESR) and c-reactive protein (CRP) were both elevated at 74 mm/h (normal male 0–20 mm/hr) and 76.9 mg/L (normal male <10 mg/L), respectively. He was started on intravenous (IV) vancomycin and cefepime and his ibrutinib was held.

The next day he underwent left knee incision and drainage with synovectomy and exchange of his tibial polyethylene component with orthopedic surgery with intraoperative specimens sent off for Gram stain and cultures. Forty-eight hours post-operative fungal stains turned positive for mold, and he was started on IV liposomal amphotericin B 5 mg/kg daily and antibiotics were discontinued.

Patient reported he was originally born in Southern California and lived there for 25 years. In his early 20's, he had acute onset of left knee pain and swelling, underwent left knee aspiration, and was told he had “Valley Fever” in his knee. Records from 1970s were not available; however, as per the patient, he was given 2 intra-articular injections followed by one year of antifungal treatment with complete resolution of his symptoms. He reported no problems with his knee until 2008 when he underwent left TKA for osteoarthritis. A CT scan of his chest four months prior to presentation for his CLL workup was noted to have a calcified granuloma in his left lower lobe suggestive of possible previous coccidioidomycosis inoculation.

**Figure 1:**
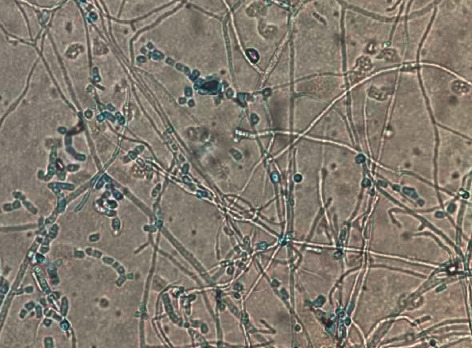
*Coccidioides immitis* under 10x magnification.

**Figure 2:**
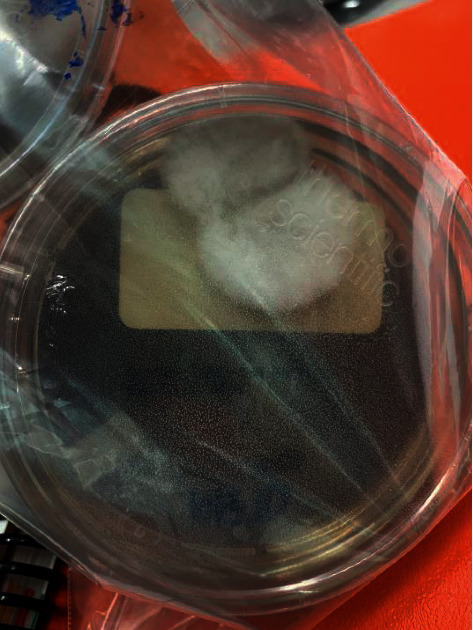
*Coccidioides immitis* gross visualization on culture media plates.

After 12 days of incubation, the cultures were visualized in the lab (Figures 1 and 2) and confirmed to be *Coccidioides immitis*. He underwent explantation of his left total knee arthroplasty with extensive synovectomy and conversion to a rigid spacer. He was switched to oral fluconazole 400 mg daily on post-operative day one after receiving 12 days of IV liposomal amphotericin B. Coccidioides complement fixation came back positive at 1:16 (normal <1:2) and patient was discharged on hospital day 15 with his ibrutinib on hold.

Six months later his left knee was re-aspirated which demonstrated no fungal growth and his ESR and CRP had also normalized. He then underwent second-stage revision of his left knee with reimplantation of hardware without complications. He is currently following the expected post-operative course and will be continued on lifelong suppressive fluconazole therapy.

## 3. Discussion

We present this case of reactivation of a latent *Coccidioides immitis* infection in a prosthetic knee 3 months after starting ibrutinib therapy for CLL/SLL in a patient who was previously treated for native knee infection 50 years ago. Host cell-mediated and adaptive immunity are required to prevent disseminated infections with *Coccidioides immitis.* Our patient experienced a two-fold disruption in these pathways with his underlying CLL/SLL, as well as with treatment with ibrutinib, predisposing him to reactivation of latent coccidioidomycosis infection in his prosthetic joint.

There have been few reports of coccidioidomycosis reactivation in the literature (Table 1), but none with a prolonged indolence of 50 years as seen in this patient. A similar scenario was described when a patient from an endemic coccidioidomycosis region moved to Spain and 30 years later underwent TKA. Then, seven years after her surgery, she had coccidioidomycosis reactivation which was treated with fluconazole suppression alone [4]. Another case series reviewed 6 cases of coccidioidomycosis infections in prosthetic joints and described their treatment regimens which included delayed two-stage arthroplasty followed by azole therapy or azole suppression alone. Two of the patients' azole therapy was stopped after 1 year and relapsed shortly after discontinuation [4]. Another unique factor in our patient's case was quick reactivation of his PJI after starting ibrutinib therapy for his CLL/SLL. Ibrutinib is a BTK inhibitor which affects neutrophil maturation and activation through inhibiting key signal cascades which directly interferes with the innate defense against fungal infections [11]. One study looked at 378 patients in which ibrutinib was used to treat leukemia where they observed 37% of patients developed an invasive fungal infection (IFI). None of these patients had the classic risk factors for IFIs which include neutropenia, lymphopenia, or high-dose corticosteroid use. Patients were at highest risk of reactivation in the first 6 months with a mean of 4.5 months. Infection rates were higher in those receiving combined corticosteroids and chemotherapy. The first infections described were invasive aspergillosis and cryptococcal meningoencephalitis followed by mucormycosis, primary central nervous system lymphoma, PJP, and even disseminated coccidioidomycosis infection [12]. It was observed that disrupting the TH2 pathway and INF-y production led to poor host fungal defense predisposing patients to IFIs.

Maintenance therapy for coccidioidomycosis infections primarily consists of the azole class of drugs that inhibits the CYP3A4 enzyme which can cause ibrutinib toxicity [11]. Under-dosing of these medications can lead to poorly controlled malignancy and high risk of infection relapse while overdosing can lead to systemic toxicities. The Infectious Disease Society of America (IDSA) recommends azole therapy for bone and joint coccidioidomycosis infections unless there is severe osseous disease or life-threatening infection, in which case amphotericin B should be utilized. Patients on biological modifiers with active coccidioidomycosis infections should be treated with azole therapy unless severe pneumonia, meningitis, or soft tissue and osseous dissemination is present, in which case liposomal amphotericin B again is recommended [12]. There are no current recommendations on which azole to use, but itraconazole is favored over fluconazole for skeletal infections, with some studies evaluating voriconazole and posaconazole use. One clinical trial involving 198 patients with various coccidioidomycosis infections involving skin, lung, and bone disease compared fluconazole versus itraconazole and found that, after 12 months, 57% responded to fluconazole at 400 mg per day and 72% of those on itraconazole 200 mg twice a day. There was no statistical superior efficacy of either treatment, but itraconazole was favored by those involved in the study [13].

Current literature suggests lifelong azole therapy, if possible, given the high risk of relapse seen in patients who stopped their azole treatment, especially in patients with a periprosthetic joint infection [5, 14]. Serial coccidioides complement fixation is a useful tool for monitoring therapeutic response [1]. Timing and utility of one versus two-stage revision surgeries is unknown, especially in the context of lifelong suppression. It is also unclear when resumption of chemotherapeutics is safe, especially given the interaction with azoles. Our patient underwent second stage of his revision 6 months after initial explant and his ibrutinib is still on hold as his CLL/SLL is stable.

In summary, this case highlights the importance of clinical history taking especially regarding infections prior to initiation of chemotherapeutics. *Coccidioides immitis* can have an extraordinary long latency period requiring clinicians to be cognizant of a patient's historical geography and raises the question for secondary prophylaxis in high-risk patients. An urgency exists for further data related to infections, specifically fungal, seen with ibrutinib.

## Figures and Tables

**Table 1:** Reported coccidioidomycosis native and prosthetic joint infections and their treatments.

Source	Case	Site of infection	Native vs. prosthetic	Type of surgery	Treatment course	Treatment response
Taxy et al. [6]		Ankle	Native	None	Oral fluconazole × 14 months	Successful
Ellerbrook et al. [7]		Knee	Native	Debridement	Oral fluconazole 600 mg/d indefinitely	Successful
Weisenberg [8]		Knee	Native	Debridement, extensive synovectomy, and meniscectomy	Oral flucaonazole 800 mg/d × 5 months and then itraconazole 200 mg/d indefinitely	Successful
Arbeloa-Gutierrez et al. [4]		Knee	Prosthetic	Prosthesis removal with amphotericin spacer and knee fusion	Oral itraconazole indefinitely	Successful
Austen et al. [9]		Knee	Prosthetic	None	Oral fluconazole 800 mg/d × 4 months and then 400 mg/d indefinitely	Successful
Kuberski et al. [5]	1	Knee	Prosthetic	Debridement, prosthesis removal, and knee fusion	Oral fluconazole 200 mg BID indefinitely	Successful
	2	Knee	Native	Synovectomy and total knee replacement	IV amphotericin (total 1000 mg) and then oral fluconazole 200 mg BID indefinitely	Successful
	3	Knee	Native	None	IV amphotericin (total 1000 mg) and then oral fluconazole 400 mg/d indefinitely	Unsuccessful
	4	Bilateral knees and ankles	Native	Right knee debridement	IV amphotericin (total 920 mg) and then oral itraconazole indefinitely	Unsuccessful
	5	Hip	Prosthetic	None	Oral fluconazole 800 mg/d indefinitely	Successful
	6	Knee	Native	Synovectomy and total knee replacement	Oral fluconazole 400 mg/d indefinitely	Unknown
Nasrawi et al. [10]		Wrist, knee, and ankle	Native	None	IV amphotericin × 12 weeks and then isavuconazonium indefinitely	Unknown

## Data Availability

Not applicable.
